# Time-course RNAseq data of murine AB1 mesothelioma and Renca renal cancer following immune checkpoint therapy

**DOI:** 10.1038/s41597-024-03294-0

**Published:** 2024-05-03

**Authors:** Wee Loong Chin, Rachael M. Zemek, Caitlin M. Tilsed, Alistair R. R. Forrest, Vanessa S. Fear, Catherine Forbes, Louis Boon, Anthony Bosco, Belinda B. Guo, Michael J. Millward, Anna K. Nowak, Richard A. Lake, W. Joost Lesterhuis, Timo Lassmann

**Affiliations:** 1 National Centre for Asbestos Related Diseases, Nedlands, WA 6009 Australia; 2 Medical School, University of Western Australia, Crawley, WA 6009 Australia; 3 University of Western Australia, Crawley, WA 6009 Australia; 4 Department of Medical Oncology, Sir Charles Gairdner Hospital, Nedlands, WA 6009 Australia; 5 Institute for Respiratory Health, Nedlands, WA 6009 Australia; 6 School of Biomedical Sciences, University of Western Australia, Crawley, WA 6009 Australia; 7 Telethon Kids Institute, University of Western Australia, West Perth, WA 6872 Australia; 8 Harry Perkins Institute of Medical Research, QEII Medical Centre and Centre for Medical Research, The University of Western Australia, Nedlands, Perth, WA 6009 Australia; 9 JJP Biologics, Warsaw, Poland; 10 Asthma and Airway Disease Research Center, University of Arizona, Tuscon, Arizona USA; 11 Department of Immunobiology, The University of Arizona, College of Medicine, Tuscon, Arizona USA

**Keywords:** Cancer immunotherapy, Cancer models

## Abstract

Time-critical transcriptional events in the immune microenvironment are important for response to immune checkpoint blockade (ICB), yet these events are difficult to characterise and remain incompletely understood. Here, we present whole tumor RNA sequencing data in the context of treatment with ICB in murine models of AB1 mesothelioma and Renca renal cell cancer. We sequenced 144 bulk RNAseq samples from these two cancer types across 4 time points prior and after treatment with ICB. We also performed single-cell sequencing on 12 samples of AB1 and Renca tumors an hour before ICB administration. Our samples were equally distributed between responders and non-responders to treatment. Additionally, we sequenced AB1-HA mesothelioma tumors treated with two sample dissociation protocols to assess the impact of these protocols on the quality transcriptional information in our samples. These datasets provide time-course information to transcriptionally characterize the ICB response and provide detailed information at the single-cell level of the early tumor microenvironment prior to ICB therapy.

## Background & Summary

Immune checkpoint blockade (ICB) has been termed the “fourth pillar” of cancer therapy^1^, but the transcriptional events underpinning treatment response remain incompletely understood. Time-critical transcriptional events in the immune microenvironment are important for response to ICB, but such events are difficult to characterise in patients^2^. For one, it is challenging to obtain repeated tumor samples from patients during treatment^3^ Furthermore, the analyses of these patient samples may be confounded by inter-individual differences in germline and cancer genetics, tumor microenvironment (TME) composition and environmental influences.

To acquire reliable tumor transcriptome data across multiple time points after ICB treatment, we created murine models with bilateral tumors derived from syngeneic cancer cell lines. In these models, ICB with antibodies against CTLA4 and PD-L1 leads to either a symmetric bilateral response or a symmetric failure to respond in both tumors^4^. This allows unilateral tumor harvest for RNA sequencing whilst allowing reliable response tracking to ICB treatment on the contralateral tumor.

**Fig. 1:**
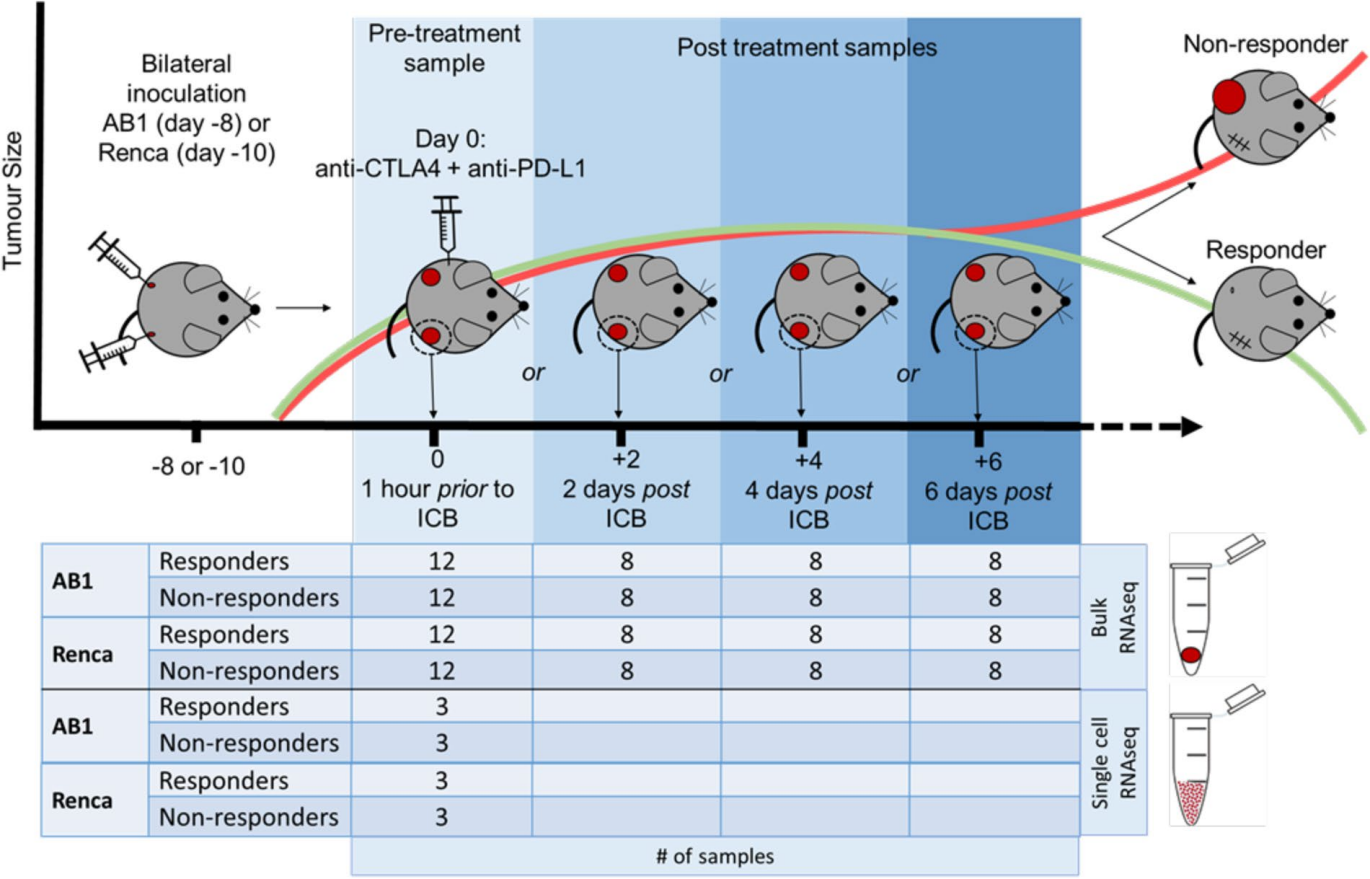
Workflow for responder/non-responder sample generation. The bilateral tumor model was used to generate samples, with tumor harvest occurring at successive timepoints pre and post treatment with ICB. Adapted from Fig. 1a in “Temporally restricted activation of IFN*β* signalling underlies response to immune checkpoint therapy in mice,” by Zemek, R. M. *et al*., Nat Commun 13, 4895 (2022).

By harvesting tumors across multiple time points in parallel experiments, we create biological replicates of reliably time-indexed RNAseq data of the tumor transcriptome, which would not be possible in a patient cohort. Previously, we used our murine models to characterize the response-associated pre-treatment TME in detail^5^. In our current work, we produced and analysed three datasets (Table 1). Firstly, we produced a set of 144 bulk RNAseq samples from AB1 mesothelioma and Renca renal cell cancer across 4 time points prior (day 0) and after treatment (day 2, 4 and 6) with ICB. Secondly, we sequenced 12 samples of AB1 and Renca tumors an hour before ICB administration using single cell sequencing (Fig. 1, Table 2). Additionally, we sequenced 4 AB1-HA tumors with bulk RNAseq that were prepared using tumor dissociation or not to determine the impact of the dissociation process on data quality. We describe this final dataset as part of our technical validation.

**Table 1:** Number of samples, timepoints, type of sequencing data available.

Sample type	Number	Description
Bulk	144	72 samples AB1, 72 samples Renca
		For each model:
		- 36 Responders and 36 NR
		- 12 time point zero, 8 time point 2,4,6
Single cell	12	6 samples AB1, 6 samples Renca
		For each model:
		- 3 responders and 3 non responders
Bulk	8	8 samples AB1-HA:
		- 4 tumor parts immediately submerged in RNAlater for bulk RNAseq
		- 4 tumor parts from same tumors gentleMACs dissociated before bulk RNAseq

**Table 2:** Sample metadata for single cell samples.

Sample name	tumor type	ICB Response
NKD180900300	Renca	Non-responder
NKD180900301	Renca	Responder
NKD180900302	AB1	Responder
NKD180900303	AB1	Non-responder
NKD180900304	AB1	Non-responder
NKD180900305	Renca	Responder
NKD180900306	Renca	Non-responder
NKD180900307	AB1	Responder
NKD180900308	AB1	Non-responder
NKD180900309	Renca	Responder
NKD180900310	Renca	Non-responder
NKD180900311	AB1	Responder

Previous datasets in the ICB literature have focused on genomic correlates with non-temporal transcriptional determinants^5,6^, fewer time points^7^ or sparser sampling density with fewer biological replicates^8^. To our knowledge, our work represents the first example of densely time-indexed RNAseq data of the early TME in tumors using both bulk and single cell RNA sequencing. In addition, it samples whole tumors prior to therapy, and compares complete responders and non-responders in the same model. We thus provide a detailed picture of the tumor transcriptome in the early stages after ICB therapy.

## Methods

With the exception of the tumour dissociation protocol described below, these methods have been described in previous work^6^. Here, we restate descriptions of the experimental murine models, cell culture techniques, *in vivo* treatments and tumour preparation for ease of reference.

### Mice

Balb/cAusB mice 8–12 weeks of age were used for all experiments. BALB/cArc mice were obtained from the Animal Resource Centre (Murdoch, WA). All mice were housed at the Harry Perkins Institute of Medical Research Bioresources Facility North under specific pathogen free conditions. Mice were kept in individually ventilated cages (Techniplast, Italy) supplied with filtered air. Cages contained aspen chips bedding (Tapvei, Estonia) and for environmental enrichment were supplemented with wood blocks, tissues, cardboard rolls and were changed every 14 days. Mice were fed Rat and Mouse cubes (Specialty Feeds, Glen Forrest, Australia) and had access to water *ad libitum*. Mice were housed at 21–22 °C with 12-hour light/dark cycle (06:00–18:00). To check SPF conditions were maintained in the animal facility, sentinel mice (n = 3) were screened for a standard panel of bacteria and fungi, non-pathogenic protozoa, endoparasites, ectoparasites and viruses (Cerberus Sciences, Australia) each month. All experiments were conducted in compliance with the institutional guidelines provided by the Harry Perkins Institute for Medical Research animal ethics committee (approval number AE047).

### Cell culture

Cell line AB1 was obtained from CellBank Australia. Cell line Renca^9^ was kindly donated by Dr E. Sotomayor and Dr F. Cheng (University of South Florida, Tampa, FL). Cell lines were maintained in RPMI 1640 supplemented with 20 mM HEPES (Gibco), 0.05 mM 2-mercaptoethanol (Sigma Aldrich), and 10% FCS (Invitrogen, Mulgrave, Australia). AB1 and Renca were supplemented with 100 units/ml penicillin/streptomycin (Thermo Fisher),. AB1-HA was supplemented with 100 U/ml benzylpenicillin (CSL), 50 ug/ml gentamycin (David Bull Labs) and 50 mg/ml geneticin (G418; Life Technologies). Cells were grown to 70–80% confluency before each passage and passaged 3–5 times before inoculation. Cells were frequently tested for mycoplasma by PCR and remained negative. Cell lines were validated yearly by flow cytometry for MHC class I molecules H2-Kb (negative) and H2-Kd (positive), and for fibroblast markers E-cad, EpCam and PDGFR*α* (negative) and by PCR for mesothelin (positive for AB1, negative for Renca).

### *In vivo* treatments

We have described the dual tumor model including inoculation and surgery in detail previously^4^. When cell lines were 70–80% confluent, they were harvested and washed 3 times in PBS. 5 × 105 cells in 100 *μ*l were inoculated subcutaneously (s.c.) onto the lower flank on both sides using a single 26 G needle per injection. Mice were randomized when tumors became palpable, approximately 3–5 days after tumor inoculation. AB1-HA was injected subcutaneously on one flank.

The anti-CLTA4 hybridoma (clone 9H10) and anti-PD-L1 hybridoma (clone MIH5) were cultured in IMDM containing 1% of FCS and gentamycin at Bioceros (Utrecht, The Netherlands). Clarified supernatants were used to purify the antibody using affinity chromatography. The antibodies were sterile formulated in PBS. Mice received an intraperitoneal (i.p.) dose of 100 *μ*g of anti-CTLA4 and 100 *μ*g anti-PDL1 combined in 100 *μ*l PBS. Mice received additional doses of 100 *μ*g anti-PDL1 two and four days later.

### Surgery experiments (responder/nonresponders)

For the pre-treatment samples, tumors were resected when they were 9 mm2, (eight days for AB1 or 10 days for Renca post tumor inoculation), and mice were administered ICB 1 hour after surgery. For the post-treatment samples, tumors were resected 2, 4 or 6 days after the first administration of ICB. Mice were dosed with 0.1 mg/kg buprenorphine in 100 *μ*l s.c. (30 min prior) and anesthetized using isoflurane (4% in 100% oxygen at a flow rate of 2 L/min). Whole tumors and the corresponding draining inguinal lymph node on the right-hand side were surgically removed and immediately immersed in RNAlater (Life Technologies, Australia). The wound was closed with staples (Able Scientific, Australia), and mice were placed in a heat box at 37 °C for recovery. As an indicator of response for the removed tumor, the remaining tumor was monitored for response. Mice were designated as responders if their tumor completely regressed and the tumor did not return for up to 4 weeks after treatment. Mice were designated as non-responders if their tumors reached 100 mm2 within 4 weeks after start of treatment, similar to saline-treated controls. Mice were designated as intermediate responders if they had a delay in tumor growth or partial regression and excluded from the analysis. For internal consistency, we only used experiments in which both responders and non-responders were present in any cage, i.e., there had to be at least one non-responder amongst responders or vice versa.

### Tumor preparation for bulk RNA sequencing

Whole tumors once surgically resected, had the surrounding tissue (e.g. skin, fat) removed and were immediately submerged in RNAlater (Life Technologies, Australia). Samples were stored at 4 °C for 24 hours, after which the samples were blotted dry and transferred to a new tube and stored at −80 °C. Frozen tumors were dissociated in Trizol (Life Technologies, Australia) using a TissueRuptor (QIAgen, Australia). RNA was extracted using chloroform and the aqueous layer was purified on RNeasy MinElute columns (QIAgen, Australia). RNA integrity (RIN score > 8) was confirmed on the Bioanalyzer (Agilent Technologies, USA). Library preparation and sequencing (50 bp, single-end) was performed by Australian Genome Research Foundation using Illumina HiSeq standard protocols.

### Tumor preparation for single cell RNA sequencing

For single-cell RNAseq, tumors were surgically resected as outlined above, and submerged in cold PBS. To obtain a single-cell suspension, tumors were cut into 1- to 2-mm pieces with a scalpel blade and dissociated using the gentleMACS system (Miltenyi Biotec). Single-cell suspensions were stored at −80 °C in 10% dimethyl sulfoxide until it could be determined if they could be designated as responders or non-responders and then they were processed for single-cell profiling. Cryo-stored cells were rapidly thawed and diluted in PBS and pelleted. Pellets were resuspended in PBS and passed through a 40-µm filter to remove cell clumps. Approximately 5000 cells per sample were then loaded onto a 10x Genomics Chromium Controller to generate Chromium Single Cell 3′ Libraries. Sequencing was carried out by Novogene Co., Ltd.

### Comparison of tumor dissociation protocols

To compare bulk vs dissociated sample gene expression, tumors were harvested and cut in half. One half was submerged in RNAlater, and the other was dissociated as described above, pelleted, and resuspended in RNAlater. Samples were stored at 4 °C for 24 hours. RNAlater was removed from bulk samples via blotting and single-cell samples by pelleting, before storing at −80 °C. Both sample types had RNA extracted as described above. Library preparation and bulk RNA sequencing (50 bp, single-end) was performed by Australian Genome Research Facility, using Illumina HiSeq standard protocols.

#### Alignment and differential expression of bulk RNAseq data

We processed a total of 144 RNAseq single-end 50 bp read samples, which comprised of four time points in two mouse models. After reviewing quality control on all samples using FastQC software, we used Kallisto (v0.43.0) for transcript abundance estimation. Gencode M21 (GRCm38.p6) transcript sequences were used to create the index for pseudoalignment. For the quantification of single-end reads, we specified an average fragment length of 200 base pairs and a standard deviation of fragment length of 20.

We also processed a further 8 RNAseq single-end 50 bp read samples for the comparison of whole versus dissociated samples using the steps described above. To analyse bulk RNAseq samples from AB1-HA prepared using gentleMACS vs RNAlater, we used the same alignment strategy with Kallisto. Following this, we performed differential expression analysis using the Wald test with Sleuth (v0.29.0), with sample preparation as a covariate. Genes were deemed differentials expressed at an absolute log fold change of 0.5 and a false discovery rate of less than or equal to 0.05. Enrichment of differentially enriched gene sets was performed with Metascape^10^.

#### Single cell sample demultiplexing and UMI counting

For single cell analysis, we processed FASTQ files from 6 AB1 and 6 Renca samples using cellranger v3.0 (10X genomics). For each sample, we performed demultiplexing and read alignment using the cellranger count function, using cellranger’s pre-supplied mm10 reference with an expect-cells parameter of 6000.

## Data Records

All sequencing data have been uploaded to the NCBI GEO database. Project data is available through accession number GSE153943^11^.

## Technical Validation

### QC control on bulk RNAseq data

**Fig. 2:**
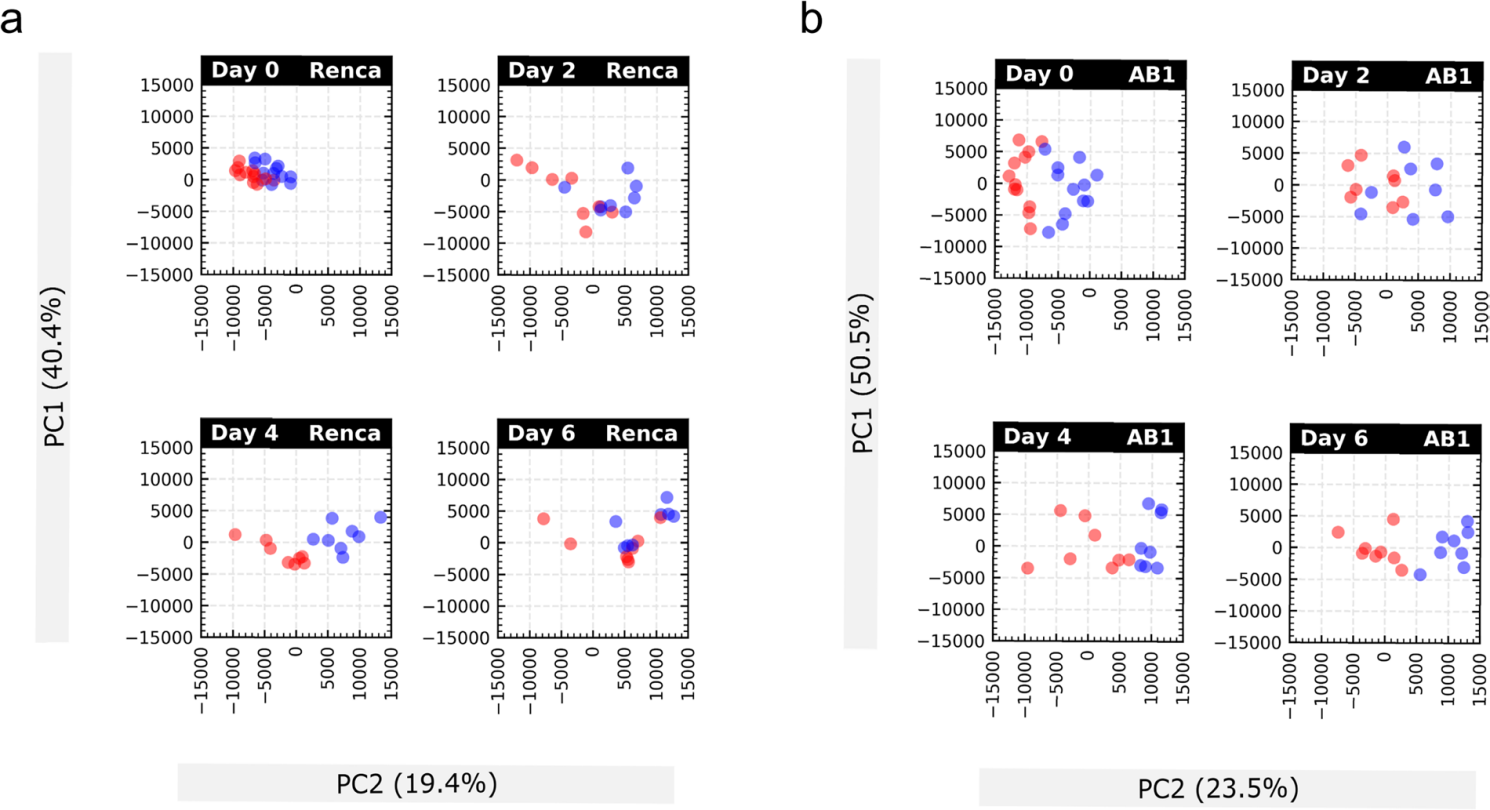
PCA plots of AB1 and Renca faceted by day and response. (**a**) shows PCA plots in Renca and (**b**) in AB1 (Blue dots represent responders; red dots non responders).

We performed FastQC across all bulk RNAseq samples sequencing depth ranging 20 −30 million reads on all samples, with approximately 40% unique reads per sample. Following alignment and transcript quantification, samples visualised with PCA (Fig. 2) did not demonstrate significant sample-specific batch effects.

### Cell labelling

**Fig. 3:**
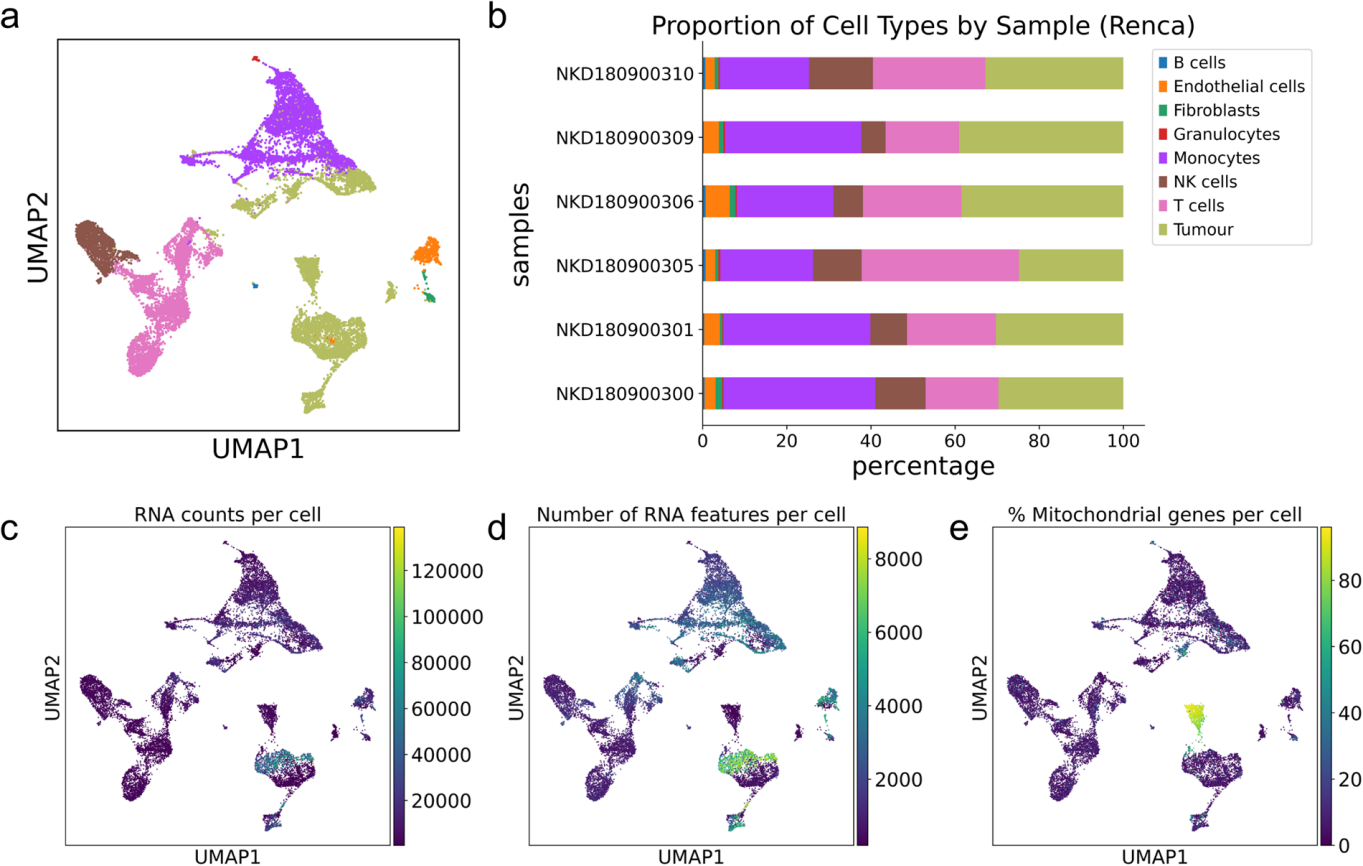
Quality control metrics for single cell data from Renca samples. (**a**) Combined Renca data from 6 single cell samples on UMAP and (**b**) sample composition for each Renca sample, faceted by cell type (**c**–**e**). Quality control metrics (RNA counts per cell, feature counts per cell and percentage mitochondrial content distribution) across all cells on Renca UMAP.

**Fig. 4:**
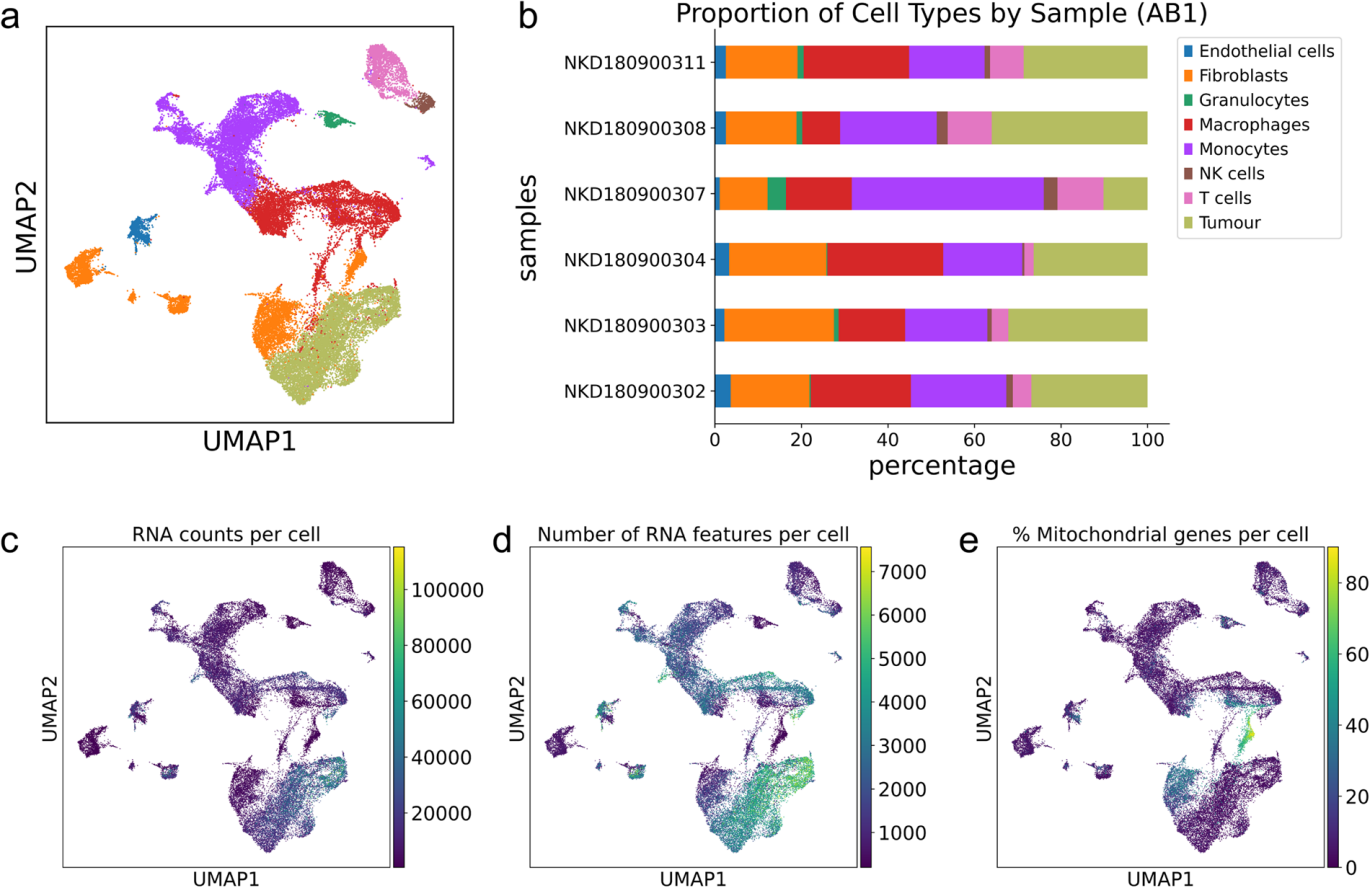
Quality control metrics for single cell data from AB1 samples. (**a**) Combined AB1 data from 6 single cell samples on UMAP and (**b**) sample composition for each AB1 sample, faceted by cell type (**c**–**e**). Quality control metrics (RNA counts per cell, feature counts per cell and percentage mitochondrial content distribution) across all cells on AB1 UMAP.

We used an automated labelling strategy based on bulk RNAseq references, which we have described in detail in a previous publication. Briefly, the R package SingleR^12^ was used in “cluster mode” using species-specific annotation references provided with the package. For annotation of murine data, we used the mouse RNAseq dataset^13^. Clusters were defined from Seurat’s FindClusters function at default (0.8) resolution. Similarly, labelled clusters were merged (Figs. 3a,b, 4a,b). We confirmed that this approach was robust to cluster size by showing that labels were consistent even when cluster size was modified by changing resolution parameters in the FindClusters function and that labelling was consistent with cell canonical markers. Since batch specific differences could introduce issues with cell identification, the consistency of cell labels allowed us to confirm the robustness of our clustering and batch-effect removal strategy.

### Normalisation and removal of batch effects

In both AB1 and Renca, we used the Seurat (version 3.14) R package to combine samples for downstream analysis. Gene counts were normalized against both sequencing depth and against the percentage of mitochondrial DNA in each cell using negative binomial regression. The resulting Pearson residuals from these processing steps were used for downstream PCA, cluster identification and UMAP embedding and visualization.

**Fig. 5:**
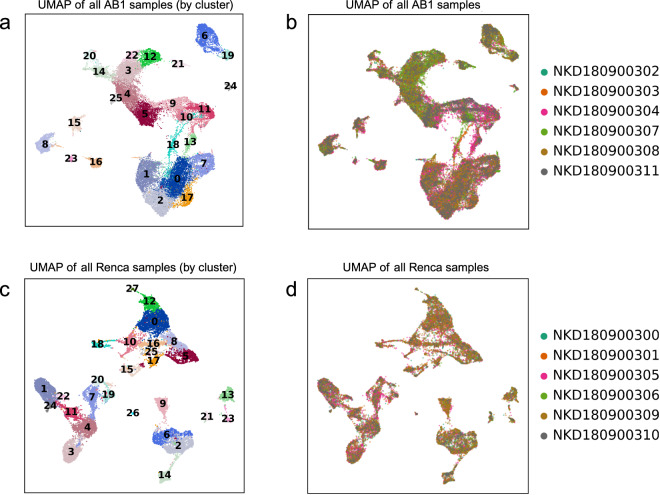
Clustering and batch effect correction on single cell data from AB1 and Renca. (**a**) UMAP of AB1 cells faceted by Seurat clusters and by (**b**) sample of origin. (**c**) UMAP of Renca cells faceted by Seurat clusters and by (**d**) sample of origin. Sample metadata is provided in Table 2.

The inspection of sample-specific UMAP plots (Fig. 5) showed that this normalisation procedure abolished batch-specific differences in AB1 and Renca samples. After QC normalisation and integration, we used 38, 000 cells in AB1 and 18,000 cells in Renca for downstream analysis. We could identify 28 cell clusters that consisted of cells in the range of 58–1877 cells per cluster in Renca and 26 cell clusters in AB1 that consisted of cells in the range of 204–3963 cells per cluster. We did not detect batch specific differences in mitochondrial content or feature count after integration with Seurat (Figs. 3c–e, 4c–e).

### Validation of the single cell RNAseq (gentleMacs) dissociation protocol

**Fig. 6:**
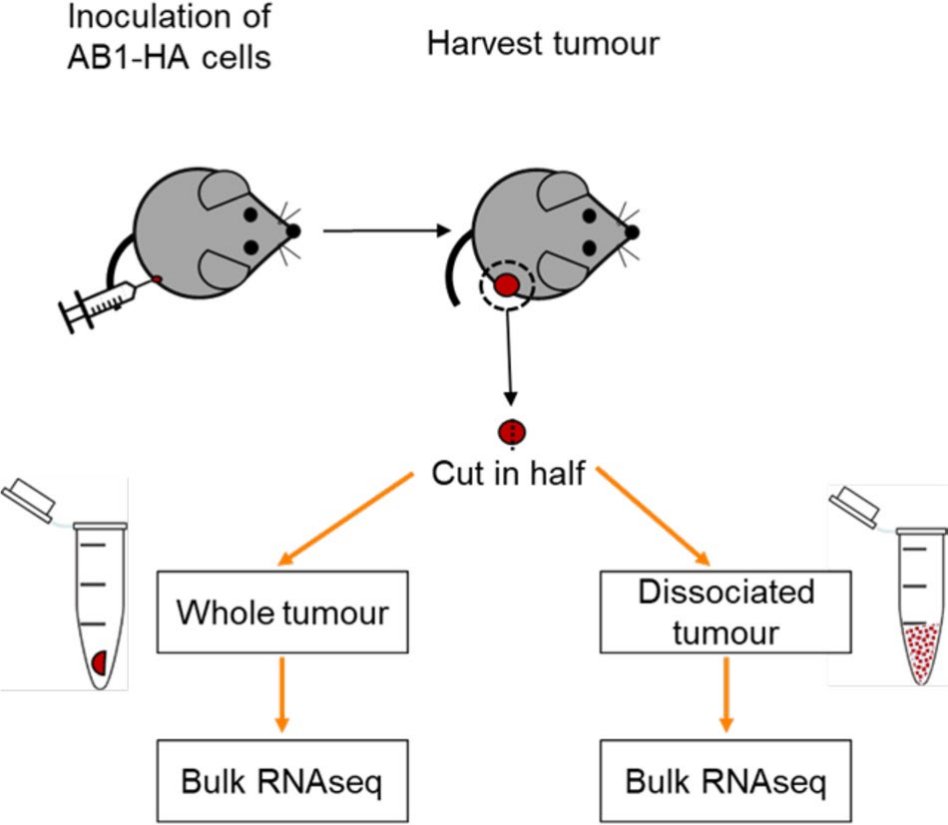
Workflow for whole vs single cell sample generation Each half was subjected to two different sample preparation protocols. Bulk RNA sequencing was performed on both sample preparations to compare the effect of these protocols on downstream differential analysis.

**Fig. 7:**
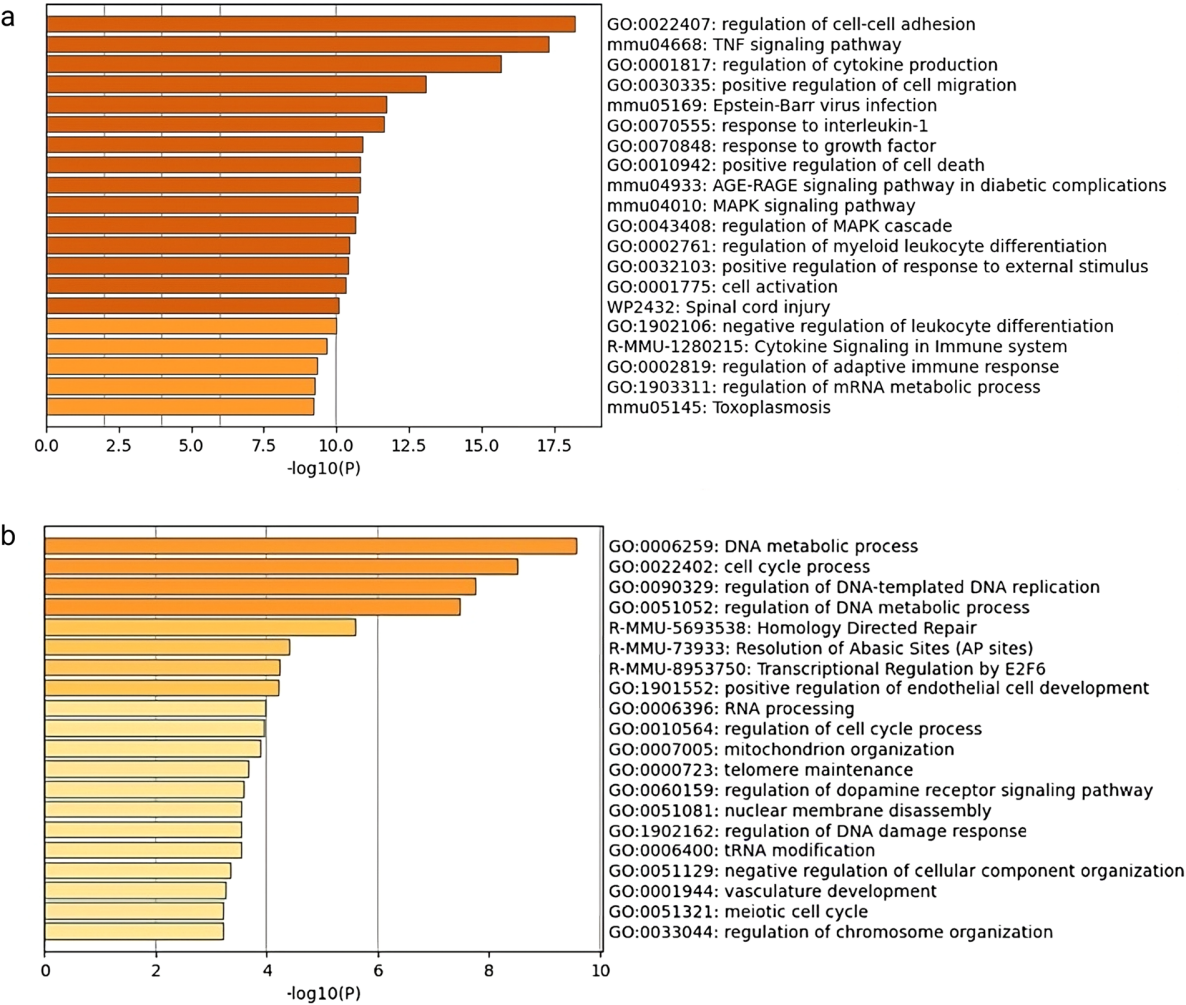
Functional annotation of DE genes between two sample dissociation protocols (gentleMACs versus maceration of frozen tissue). (**a**) functional annotation for upregulated genes after dissociation using gentleMACs. (**b**) functional annotation downregulated genes using gentleMACs.

Processing of bulk RNA seq samples involved disruption of RNAlater-preserved frozen tissue in Trizol. In contrast, the single cell sample preparation involves a cell dissociation step at room temperature using the gentleMACs protocol. To assess the impact of these protocols on the quality transcriptional information in our samples, we performed bulk RNA sequencing on 8 samples, with 4 samples prepared using either protocol (Fig. 6). We performed downstream analysis with DE and pathway analysis. In samples prepared using gentleMACS dissociation, 871 DE genes were upregulated and 840 genes downregulated relative to dissociation of frozen macerated tissue in Trizol (thresholded at log-fold change (beta-value) of 1.0, fdr level of 0.05). Pathway analysis using metascape^10^ on these differentially expressed genes is shown in Fig. 7.

## Data Availability

The code used to reproduce these analyses are available on GitHub https://github.com/wlchin/scientific_data_ab1_renca as snakemake^14^ workflows.
